# Predator strike shapes antipredator phenotype through new genetic interactions in water striders

**DOI:** 10.1038/ncomms9153

**Published:** 2015-09-01

**Authors:** David Armisén, Peter Nagui Refki, Antonin Jean Johan Crumière, Séverine Viala, William Toubiana, Abderrahman Khila

**Affiliations:** 1 Institut de Génomique Fonctionnelle de Lyon, CNRS-UMR5242, Ecole Normale Supérieure, Université Claude Bernard, 46 Allée d’Italie, 69364 Lyon Cedex 07, France

## Abstract

How novel genetic interactions evolve, under what selective pressures, and how they shape adaptive traits is often unknown. Here we uncover behavioural and developmental genetic mechanisms that enable water striders to survive attacks by bottom-striking predators. Long midlegs, critical for antipredator strategy, are shaped through a lineage-specific interaction between the Hox protein Ultrabithorax (Ubx) and a new target gene called *gilt*. The differences in leg morphologies are established through modulation of *gilt* differential expression between mid and hindlegs under Ubx control. Furthermore, short-legged water striders, generated through *gilt* RNAi knockdown, exhibit reduced performance in predation tests. Therefore, the evolution of the new Ubx–*gilt* interaction contributes to shaping the legs that enable water striders to dodge predator strikes. These data show how divergent selection, associated with novel prey–predator interactions, can favour the evolution of new genetic interactions and drive adaptive evolution.

## Abstract (web summary)

Understanding the mechanism underlying the evolution of ecologically relevant traits is challenging. Here the authors show that changes in the Hox protein Ultrabithorax and its target gene *gilt* contribute to the evolution of long-mid-legs in water striders, a critical trait to escape predators.

## Graphical abstract

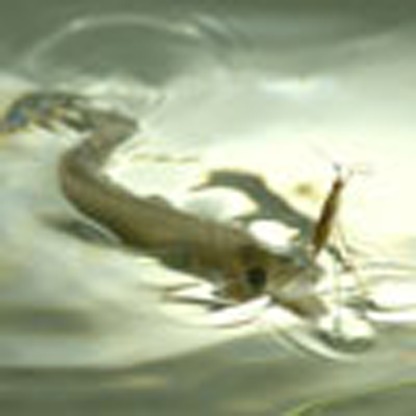

The discovery that distinct lineages share a similar genetic toolkit established that variations within genetic networks could shape the phenotype during development and evolution^1^,^2^. An important gap in understanding the origin of phenotypic diversity, however, resides in our poor knowledge about how selection can favour the emergence of novel developmental genetic interactions and ultimately shape phenotypic evolution^3^,^4^,^5^. Here we focus on the semi-aquatic bugs (Heteroptera, Gerromorpha) to study how divergent selection, associated with a new lifestyle on the water surface^6^, can drive species diversification. The ancestors of the Gerromorpha transited from terrestrial life to life on the water surface over 200 Myr ago^6^,^7^,^8^. Acquisition of this lifestyle exposes the insects to new challenges including locomotion on fluid and escaping bottom-striking predators. Paramount to their success on water surfaces is the evolution of the shape and size of their legs in association with niche specialization and mode of locomotion^6^,^9^,^10^. Basal lineages, which occupy transitional zones between water and land, retain ancestral character states with midlegs shorter than hindlegs, and employ the ancestral mode of locomotion through alternating leg movements^6^,^7^,^9^,^11^,^12^. In contrast, water striders (Gerridae) that specialize in open water zones employ a derived mode of locomotion through simultaneous rowing motion of the midlegs. This rowing mode is enabled by a novel character state where midlegs are now longer than hindlegs^6^,^11^,^12^,^13^. A hallmark of the entire lineage of the semi-aquatic bugs, that distinguishes them from closely related terrestrial Heteroptera, is the deployment of the Hox protein Ubx in the second thoracic segment^11^,^13^ and a notable increase of Ubx dose in the third thoracic segment^12^. These changes in Ubx expression and function are associated with changes in leg length across the semi-aquatic bugs^11^,^12^,^13^. While we are beginning to understand how morphological changes in the appendages are associated with specificities of locomotion and sexual interactions on the fluid water–air interface^6^,^10^,^14^, little is known about how the legs are shaped under selection by aquatic predators. Here we investigated the evolutionary and developmental genetic mechanisms underlying antipredator strategies in water striders. We found that water striders survive attacks by aquatic predators using a quick jump enabled by their long and slender legs. We demonstrated that the shape of the legs, that are important for this behaviour, is modulated through the evolution of a lineage-specific interaction between Ubx and a new target gene called *gilt*. Finally, we showed that short-legged individuals, generated through *gilt* RNAi, exhibit reduced jump performance in predation tests.

## Results and discussion

### Water striders use quick jump, and not flight, to escape predators

**Figure 1:**
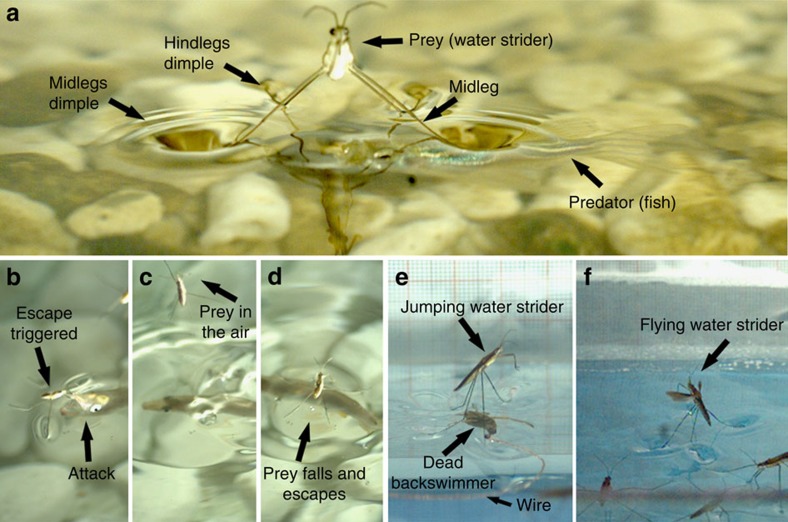
Predation escape strategy and behavioural setups in water striders. (**a**) A water strider initiating the escape through a jump triggered by the approaching fish. The midlegs push downwards causing them to bend and form deep depressions (dimples). (**b**–**d**) Frames, taken from Supplementary Movie 1, showing the sequence of interaction between the water strider and the attacking halfbeak fish. The water strider dodges the attack (**b**) by moving away from the trajectory of the fish’s strike through a quick jump in the air (**c**), while the fish is carried away by its own momentum, the water strider falls back and skates away (**d**). (**e**) Controlled set-up used to trigger the jump in adult water striders using a dead backswimmer manoeuvred by the experimentalist with a wire. (**f**) Flight in water striders triggered by increasing population density and exposure to high light intensity.

First, we asked how water striders survive in the face of the various predators lurking under the surface^15^,^16^,^17^ (Supplementary Movie 1). Using high-speed videography, we examined the interaction between the water strider *Limnoporus dissortis* and the stealth surface hunting halfbeak fish *Dermogenys sp*^18^. When attacked from beneath, the water strider uses an almost vertical jump in the air to avoid the strike (Fig. 1; Supplementary Movie 1 section 3). The animal pushes against the water surface with a characteristic bending of the mid and hindlegs, creating dimples without breaking surface tension (Fig. 1a; Supplementary Movie 1 section 3). The first legs do not seem to contribute. This behaviour results in movement amplification that propels the insect several times higher in the air than the length of its body. The time from the trigger of the strike until when the fish’s jaws reach the water strider is 28.7±3.9 ms, on average (Supplementary Table 1). As a result of the jump (Fig. 1b), the fish misses (Fig. 1c) and the water strider falls back and escapes (Fig. 1d; Supplementary Movie 1 section 3). To measure the duration of the jump, we developed a controlled set-up where the attack is simulated using a dead backswimmer (*Notonecta)*, a natural predator of water striders^17^, which we manoeuvred with a wire (Fig. 1e). This set-up reproduced the water strider jump, with 28.8±1.8 ms from when the jump is triggered until when the legs of the animal lose contact with water surface (Supplementary Movie 1 section 3; Supplementary Table 1). Therefore, the water strider delivers an antipredator response that matches the quick strike of the predator. Interestingly, we observed that adult water striders invariably jump to dodge the strike even though they are capable of flight^6^,^19^. When we triggered flight (Fig. 1f, see details in Methods and SOM), we found that the water strider takes an average of 124.8±18.6 ms to leave the water surface. Eighty per cent (99.4±18.0 ms) of the flight time is accounted for by the animal flapping its wings, while the remaining twenty per cent (25.4±12.8 ms) represent take-off supported by the legs and the wings (Supplementary Movie 2). This indicates that flight is four times significantly slower (one-way analysis of variance (ANOVA), *n*=10, *P*<0.0001) than the jump, that is, four times slower than the predatory strike, and thus not suitable as an antipredator strategy (Supplementary Table 1; Supplementary Movie 2). Therefore, the jump enabled by the slender long legs is critical for the water strider to survive aquatic predators.

### Emergence of novel gene expression in water striders

**Figure 2:**
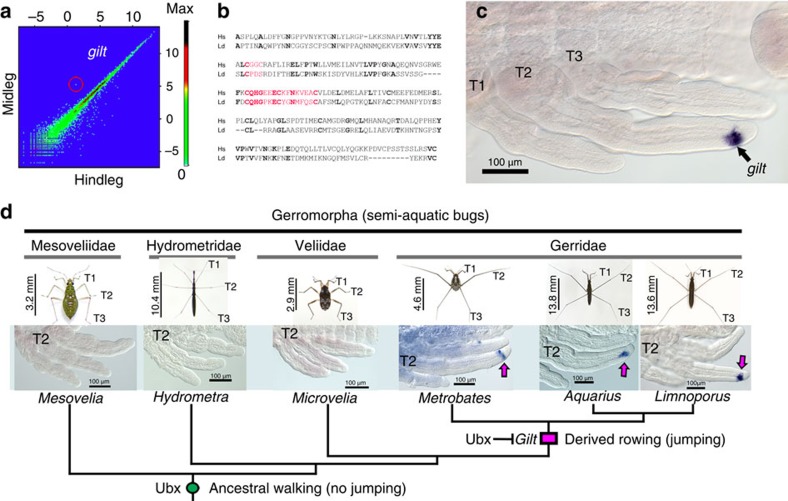
*gilt* expression during development and evolution in the semi-aquatic bugs. (**a**) Comparative analysis of gene expression profiles between midlegs and hindlegs during early embryogenesis in *Limnoporus dissortis*. The transcript corresponding to *gilt* is encircled. (**b**) Sequence comparison between *L. dissortis* and *Homo sapiens* Gilt proteins. Gilt active site and signature motif are highlighted in red, identical amino acids are in bold. (**c**) *In situ* hybridization detecting *gilt* mRNA expression in the midlegs of an early *Limnoporus* embryo (48 h: ∼30% development). T1, 2, 3: Thoracic segments 1, 2, 3. (**d**) Evolution of *gilt* expression across six semi-aquatic bugs representing four families and both the ancestral and derived leg-length plan. *gilt* expression in the midlegs is detected in species with derived relative leg length plan and absent in species with the ancestral leg plan. The gain of Ubx in midlegs at the base of the Gerromorpha lineage is indicated with green circle and the evolution of *gilt* expression at the base of the Gerridae with pink rectangle. Scale bars indicated are body sizes of adult individuals.

The derived morphology where midlegs are longer than hindlegs is important for this antipredator behaviour. We therefore wanted to uncover the genetic mechanisms that shape the differences between the legs during development in water striders. A genome-wide comparative analysis of gene expression profiles uncovered 155 differentially expressed transcripts between midlegs and hindlegs of developing *Limnoporus* embryos (Fig. 2a). We further focused on a transcript the expression of which perfectly matched the differences in morphology between the legs, that is, high in the midlegs and very low in the hindlegs (Fig. 2a; Supplementary Fig. 1a–c). This transcript is homologous to a gene known as *gamma interferoninducible thiol reductase* (*gilt*) (Fig. 2b), a reducing enzyme required for antigen processing and presentation by the major histocompatibility complex II in mammals^20^. A role for Gilt in innate immunity has recently been suggested in flies^21^, but no known developmental function has ever been associated with this protein. During water strider development, we found that *gilt* mRNA expression is specific to the midlegs. Its expression begins at ∼25% of embryogenesis (Supplementary Fig. 1d), exclusively in a distinct cell population at the tip of midleg tarsi, and continues through later stages of embryogenesis (Fig. 2c; Supplementary Fig. 1e,f). *gilt* is exclusively expressed in the midlegs until 45% of embryogenesis, but also appears in the hindlegs during later embryogenesis (Supplementary Fig. 1b,c). While all Gerridae have the derived leg plan where midlegs are longer than hindlegs, many other Gerromorpha retain the ancestral leg plan where midlegs are shorter than hindlegs^6^,^7^,^11^,^12^. We therefore cloned *gilt* from a sample of six species representing the two morphologies (Supplementary Fig. 1g). We found that *gilt* is expressed in the tarsus of midlegs in all three species with derived leg plan and absent from all three species that retain an ancestral leg plan (Fig. 2d). This indicates that the expression of *gilt* in the midlegs is characteristic to the derived morphology and that its evolution coincides with the evolution of rowing characteristic of species specialized in open water zones.

### Ubx modulates *gilt* expression in the legs

**Figure 3:**
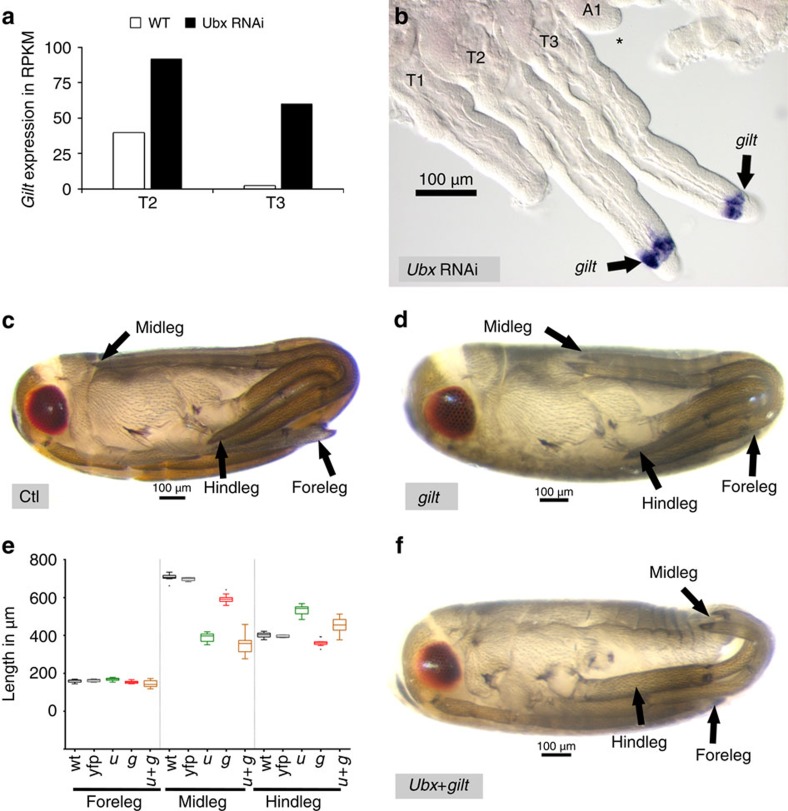
*gilt* function and interaction with Ubx in *Limnoporus dissortis*. (**a**) Transcriptomics data show that *gilt* expression doubles in the midleg and appears in the hindlegs of *Ubx* RNAi embryos, as revealed by the number of reads per kilobase per million (RPKM). (**b**) *In situ* hybridization detecting *gilt* mRNA in an *Ubx* RNAi early embryo. Note the expansion of *gilt* in the midlegs and its ectopic expression in the hindleg. T1, T2 and T3: Thoracic segments 1, 2 and 3; A1: Abdominal segment 1; Asterisk (*) indicates an ectopic leg forming on A1 and that is characteristic to *Ubx* RNAi in water striders^12^. (**c**) Control late embryo. (**d**) *gilt* RNAi late embryos with shorter midlegs. (**e**) Tukey box plots showing measurements of the effect of *u* (*Ubx* RNAi *n*=13), *g* (*gilt* RNAi *n*=12), or *u+g* (double *Ubx*+*gilt* RNAi *n*=21) RNAi on leg length compared with controls (wt untreated *n*=10 and injected with YFP double-stranded RNA *n*=7). (**f**) Double *Ubx*+*gilt* RNAi late embryo showing drastically shorter midlegs. Scale bars are indicated.

Previous work has shown a role for Ubx in the acquisition of the novel long midleg body plan, with moderate levels of Ubx lengthening midlegs and six to seven times higher levels of Ubx shortening hindlegs^11^,^12^,^13^. Interestingly, our comparative transcriptome shows an inverse correlation between *Ubx* and *gilt* levels—with low *Ubx* high *gilt* in midlegs, and high *Ubx* low *gilt* in hindlegs (Supplementary Fig. 2) suggesting a regulatory interaction between Ubx and *gilt*. To test this possible interaction, we contrasted a transcriptome of legs extracted from *Ubx* RNAi embryos to that of legs from untreated embryos (Fig. 3a). We found that in *Ubx* RNAi embryos *gilt* expression doubles in the midlegs and appears ectopically in the hindlegs (Fig. 3a). Furthermore, *in situ* hybridization in embryos treated with *Ubx* RNAi confirms the appearance of *gilt* expression in the hindlegs (Fig. 3b), demonstrating that *gilt* is no longer repressed when we knock *Ubx* down. Therefore, Ubx represses *gilt* partially in the midlegs and entirely in the hindlegs of *Limnoporus* early embryos, and the extent of this repression is dependent on Ubx levels.

### Gilt is required to lengthen the midlegs in water striders

We further tested the role of *gilt* during embryonic leg development using parental RNA interference^11^,^12^,^13^. Embryos with depleted *gilt* transcript (*n*=12) grow significantly shorter midlegs relative to controls (YFP-injected controls *n*=7 and untreated controls *n*=10, one-way ANOVA *P*<0.0001; Fig. 3c,d) (The various controls for this experiment are detailed in the Methods and Supplementary Fig. 3). Statistical analyses of measured leg length revealed that *gilt* RNAi embryos develop significantly (12%) shorter midlegs compared with controls (Fig. 3e). This shortening affects the tarsus, tibia and femur (Supplementary Fig. 4), suggesting that the role of *gilt* in leg allometry is cell non-autonomous. *gilt* expression increases in *Ubx* RNAi treatments (Fig. 3a), whereas either *Ubx*^11^,^12^,^13^ or *gilt* RNAi results in shorter legs (Fig. 3d,e). To test whether increased *gilt* expression impacts leg length in the absence of Ubx, we performed a double *Ubx+gilt* RNAi experiment (Fig. 3e,f; details and controls can be found in Methods and Supplementary Fig. 5). Midlegs in double *Ubx+gilt* RNAi (*n*=21) are 7% significantly shorter (one-way ANOVA, *P*<0.0046) than midlegs of single *Ubx* RNAi (*n*=13; Fig. 3e). Conversely in the hindlegs, double *Ubx+gilt* RNAi produces an intermediate effect between single *gilt* (one-way ANOVA, *P*<0.0001) and single *Ubx* (one-way ANOVA, *P*<0.0001) RNAi (Fig. 3e). Therefore, part of the role of Ubx in lengthening the midlegs allows some expression of *gilt*, and part of its role in shortening the hindlegs requires the complete silencing of *gilt*. Altogether, our data demonstrate that *gilt* represents a downstream effector of Ubx, whose expression contributes to the long leg phenotype that is associated with the evolution of the antipredator jump behaviour.

### Gilt-free water striders jump lower

**Figure 4:**
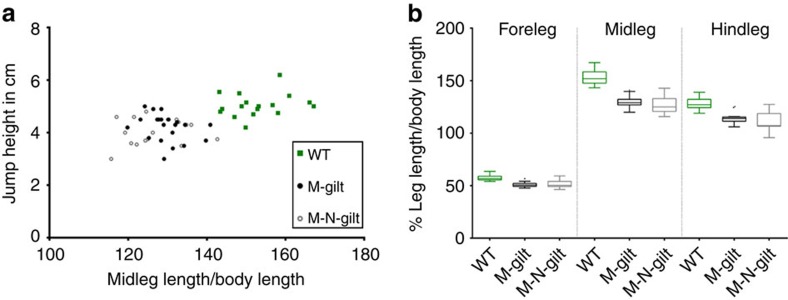
Decreased performance of *gilt* RNAi water striders in predation test. (**a**) Tukey box plots showing leg lengths variations within normal and *gilt* RNAi individuals at the fifth nymphal instar used in this test. Wild types (*n*=17) are untreated, M-*gilt (*Maternal *gilt* RNAi*; n*=21) are fifth nymphal instar individual obtained by injecting mothers with *gilt* ds-RNA, and M–N-*gilt* (Maternal plus Nymphal *gilt* RNAi; *n*=17) received an additional injection at first nymphal instar. (**b**) Lower performance in simulated predation tests directly correlates with midleg length reduction across *gilt* RNAi individuals.

Unlike Ubx, the depletion of *gilt* does not impair whole-body development and instead allows the emergence of viable adults without any apparent defects other than the reduction of leg length. *gilt* RNAi animals therefore provided an exquisite opportunity to directly assay the importance of leg length for antipredator strategy. To do this, we measured the jump performance of water striders when attacked from beneath using our controlled set-up (Fig. 4). On average, control individuals (*n*=17) jumped 5.05±0.46 cm (Fig. 4a), while *gilt* RNAi individuals (*n*=38) jumped 4.13±0.51 cm (Fig. 4a), that is, a performance 18% significantly lower than that of normal individuals (one-way ANOVA, *P*<0.0001; Supplementary Movie 3). Interestingly, *gilt* RNAi animals display a varying degree of leg length reduction (in average 16 and 6% shorter mid and hindlegs, respectively; Fig. 4b) and there is a clear correlation between midleg length and jump performance (Fig. 4a). These results establish that the acquisition of *gilt* expression contributes to shaping the legs in association with the evolution of the jump behaviour, and that even a small reduction in leg length reduces the ability of water striders to deliver a powerful jump when the predator strikes.

Altogether, our findings show that the predation escape strategy employed by water striders relies primarily on a jump reflex, enabled by the long slender legs. We also show that the acquisition of this morphological character involves novel genetic interactions between the Hox protein Ubx and *gilt;* a previously unknown target, encoding a reducing enzyme. When compared with species that retain ancestral character states where midlegs are shorter than hindlegs, the gain of *gilt* expression contributes to increase the length of midlegs that is characteristic to species specialized in rowing in open waters. Therefore, the evolution of this novel interaction between Ubx and *gilt* contributes to shaping the legs in association with the efficient antipredator strategy that water striders employ to survive aquatic predators^22^. The jump, though representing a quick reaction to the strike, can only give advantage to the prey if coupled with a primary mechanism of predator detection^23^. Despite the stealth approach of the fish, water striders are exquisite sensors of vibration owing to a multitude of sensory bristles located in the contact surface between the legs and the water surface^24^. Therefore, the ability of water striders to detect predators, together with the adapted morphology of their legs may have been key to their success in open water surfaces worldwide. This rich ecological and evolutionary context, together with the amenability to experimental manipulation, make water striders a suitable system to study how genetic interactions evolve in association with adaptive phenotypic evolution.

## Methods

### Animal collection and rearing

*Limnoporus dissortis*, *Microvelia americana* and *Metrobates hesperius* were collected from ‘Rivière de l’Acadie,’ at the vicinity of Montréal, Québec, Canada. *Aquarius paludum* and *Hydrometra stagnorum* were collected in a pond near Lyon, France. *Mesovelia mulsanti* were collected in a pond near Cayenne, French Guiana. Halfbeak fishes (*Dermogenys sp.*) were purchased from a fish store in Lyon, France. Both water striders and fish were kept in aquaria at 25 °C with a 14-h light/10-h dark cycle, and both were fed on live crickets. Pieces of floating Styrofoam were regularly supplied to female water striders to lay eggs.

### Measurement of fish strike duration

Ten high-speed movies were captured using a Miro M310 high-speed camera (Vision Research), at 2,000 frames per second, while halfbeak attacked water striders. In each movie, we manually identified the start frame where the animal first triggered the attack and the end frame where the fish’s jaw reach the initial position of the body of the water strider. The strike duration represented by the time elapsed between the start and the end frames was calculated using PCC software (Vision Research). Details about software and method of calculation can be found in this tutorial by Vision Research: http://www.visionresearch.com/Service--Support/Tutorials/

### Measurement of water strider jump duration

This experiment was conducted in adult water striders to compare with the duration of flight below. To measure jump duration in a controlled manner, we simulated the attack using a dead backswimmer (*Notonecta)* attached at the end of a wire and manoeuvred by a manipulator. This strategy reproducibly induced water striders to jump. We then filmed ten individuals at 2,000 frames per second. In each movie, we identified the start of the jump as the time elapsed between the frame where we see the first movement of the leg pushing downwards, and the frame where all legs lose contact with the water surface. Measurements of the time elapsed between start and end of the jump were performed using PCC software (Vision Research).

### Measurement of flight duration

To measure flight duration, we stimulated flight by increasing the density of water striders in the aquaria and by exposing them to an extra source of light. These conditions originate from our own observations while breeding water striders in the lab. We then took ten high-speed movies (2,000 frames per second) of 10 individuals during flight. In each movie, we identified the start of the flight as the time elapsed between the frame where we first see wings moving and the frame where all legs lose contact with the water surface. Measurements of the time elapsed between start and end of the flight were performed using PCC software (Vision Research).

### *Limnoporus dissortis* reference transcriptome

A mixture of adult males and females, nymphal instars and various embryonic stages of *L. dissortis* were used to isolate total RNA using Trizol (Invitrogen). Transcriptome was sequenced using 454 Roche technology and assembled using Newbler program version 2.6 (Roche). A total of 26,237 isotigs defining 16,368 isogroups were assembled. Assembled isotigs were annotated by sequence similarity against the NCBI ‘non-redundant’ protein database using BLAST2GO. In addition to inferred annotations, for each annotated isogroup we predicted PFAM motifs using HMMER3 tools from Sanger institute. *L. dissortis* transcriptome can be retrieved in NCBI using the following accession number: PRJNA289202.

### Quantification of gene expression using comparative transcriptomics

RNA from dissected *L. dissortis* legs was used by *ProfilExperts* (Lyon-France) to conduct deep sequencing using TruSeq RNA kit and Illumina HiSeq2000 technology. A total of ∼50 million reads was generated per sample. These reads were trimmed and filtered to remove low-quality bases and aligned against the draft *Limnoporus* transcriptome and transcript levels were quantified by determining the number of Reads Per Kilobase per Million mapped reads (RPKM) in each sample^25^. The raw reads of *L. dissortis* leg transcriptomes can be retrieved here: PRJNA289202.

### *gilt* cloning

Total RNA from *L. dissortis, A. paludum*, *M. hesperius*, *M. americana*, *H stagnorum*, and *M. mulsanti* was extracted from different embryonic stages and nymphal instars. First strand cDNA synthesis was then performed for each species, using total RNA as a template, according to instructions outlined in the Invitrogen cDNA synthesis kit. Specific primers for *gilt* in *L. dissortis* were designed based on sequences obtained from the whole transcriptome of *L. dissortis*. Primers for each species used in PCR reaction to amplify a fragment of *gilt*, can be found in Supplementary Table 2. *gilt* sequence for each species can be retrieved in Genbank. *gilt* sequences from these various species can be retrieved in GenBank using the following accession numbers: KR704883, KR704884, KR704885, KR704886, KR704887, and KR704888.

### Embryo dissection

Embryos were collected, treated with 25% bleach, and then washed with PTW 0.05% (1 × PBS; 0.05% Tween-20). For image acquisition, late embryos were dissected out of the eggshell, fixed in 4% formaldehyde, and their images captured using a Zeiss Discovery V12 scope. For staining, embryos of various stages were manually dissected, cleaned from yolk and fixed following the method below.

### *In situ* hybridization

Dissected embryos were fixed in 200 μl 4% Paraformaldehyde (PFA)+20 μl dimethyl sulfoxide, and 600 μl heptane for 20 min at room temperature with shaking. Embryos were then washed several times in cold methanol and rehydrated in decreasing concentrations of methanol in PTW 0.05%. These embryos were washed three times in PTW 0.05%, three times in PBT 0.3% (1 × PBS; 0.3% Triton X100), and twice with PBT 1% (1 × PBS; 1% Triton X100). Following these washes, embryos were transferred to 1:1 PBT 1% / hybridization solution (50% formamide; 5% dextran sulfate; 100 μg−ml^−1^ yeast tRNA; 1 × salts). The composition for 100 ml 10 × salt is as follows: 17.5 g sodium chloride, 1.21 g tris-base, 0.71 g monosodium phosphate, 0.71 g sodium phosphate dibasic, 0.2 g Ficoll 400, 0.2 g Polyvinylpyrrolidone (PVP), 10 ml of 0.5 M EDTA, 0.2 g BSA (pH 6.8). Embryos were prehybridized for one hour at 60 °C, followed by addition of a Dig-labelled RNA probe overnight at 60 °C. Embryos were then transferred gradually from hybridization solution to PBT 0.3% through consecutive washes with 3:1, 1:1, 1:3 prewarmed hybridization solution: PBT 0.3% gradient. A blocking step was performed with PAT (1 × PBS; 1% Triton X100; 1% BSA) at room temperature followed by incubation with anti-DIG antibody coupled with alkaline phosphatase for 2 h at room temperature, or at 4 °C overnight. Embryos were washed several times in PBT 0.3% then in PTW 0.05% before colour reaction is conducted with NBT/BCIP in alkaline phosphatase buffer (0.1 M Tris pH 9.5; 0.05 M MgCl2; 0.1 M NaCl; 0.1% Tween-20).

### Parental RNAi

Gene knockdown of *Ubx* and *gilt* using parental RNAi was conducted following the protocol described in references^11^,^12^,^13^. Control RNAi was conducted by injecting ds-*yfp* (double-stranded (ds) RNA from the *yfp* gene sequence). Template for *in vitro* transcription to produce ds-RNA for each gene was prepared using the T7-tagged primers in Supplementary Table 3.

### Efficiency of *gilt* RNAi

We verify that *gilt* RNAi specifically depletes *gilt*, we stained embryos with a mix containing *gilt* probe and a 10-fold diluted *egfr* probe. *egfr* is expressed in the legs in five stripes prefiguring the joints between leg segments^12^. This probe mix allows *gilt* staining to become strong and easily visible before *egfr* staining. If our RNAi experiment successfully depletes *gilt*, then we expect to see *egfr* staining but not *gilt*. In control treatments, this probe mix stains the embryos such that *gilt* staining appears stronger than *egfr* staining, thus allowing us to assess *gilt* expression relative to the strength of *egfr* staining (Supplementary Fig. 3a). Staining embryos from *gilt* RNAi treatments with the same mix fails to detect *gilt* mRNA in midlegs tarsi even when we allow *egfr* staining to become strong (Supplementary Fig. 3b). This demonstrates that our RNAi experiment efficiently depletes *gilt* transcripts.

### Specificity of *gilt* RNAi

To ensure that our RNAi does not suffer from off target effects, we designed double-stranded RNA from two non-overlapping fragments of *gilt* (Supplementary Fig. 3c). Injection of ds-RNA from these three fragments induced the same leg length phenotypes (Supplementary Fig. 3d–f). Furthermore, we repeated the same experiment on a second species, *A. paludum*, and obtained the same results (Supplementary Fig. 3g–j). Therefore, gilt RNAi depletes specifically *gilt* transcript. To make sure that the leg-shortening phenotype of *gilt* RNAi is not just a reflexion of natural variation in leg length, we measured the legs of individual that reach the first nymphal instar at successive days (Supplementary Fig. 3k). We found that the first individual that are laid by injected females (days 1 and 2) have either normal leg length or very subtle shortening. The shortening phenotype becomes stronger the following days (Supplementary Fig. 3k). This indicates that the effect we see on leg length is due to *gilt* knockdown.

### Efficiency of double *gilt+Ubx* RNAi

We confirmed the efficiency of the double *gilt+Ubx* knockdown by staining embryos with the mix containing *gilt* probe and 10-fold diluted *egfr* probe (Supplementary Fig. 5). Control embryos show a clear *gilt* expression in the midlegs (Supplementary Fig. 5a), *Ubx* RNAi embryos show *gilt* expression in both mid- and hindlegs (Supplementary Fig. 5b), whereas single *gilt* RNAi (Supplementary Fig. 5c) and double *gilt/Ubx* RNAi (Supplementary Fig. 5d) show no *gilt* expression in either leg despite the clear *egfr* staining. This demonstrates that the double *gilt+Ubx* RNAi knockdown was successful.

### Leg measurements

A sample of first instar nymphs was used for each RNAi group and control: untreated control *n*=10; *yfp*-injected negative control *n*=7; *gilt* RNAi *n*=12; *Ubx* RNAi *n*=13; and *Ubx*+*gilt* RNAi *n*=21. Nymphs were dissected and mounted on slides in Hoyer’s medium. Three sets of measurements were recorded: head width and the length of the tarsus, tibia and femur individually. Measurements for each leg segment of each pair of legs were recorded on a Zeiss microscope using the Zen software.

### Predation test experiment

We conducted predation performance in a controlled set-up that induces the water striders to jump near a grid such that the height of the jump can be accurately measured. We triggered the jump by approaching a dead backswimmer under the water striders. Individuals from the different categories (WT, Maternal-*gilt* RNAi and Maternal–Nymphal-*gilt* RNAi) were allowed to develop until fifth nymphal instar. We used fifth instars because they are large enough for recording and also because they easily induced to jump. We took a total of seven movies for each individual and extracted jump height. We then killed the individuals and measured their leg length and body length. Finally, we plotted the highest jump from each individual by its leg length corrected to body length.

### Statistical analyses

All statistical analyses and plots were performed using GraphPad Prism (version 6.00). For differences in leg length between controls and the various RNAi experiments (*gilt, Ubx, Ubx+gilt*), we performed a one-way ANOVA test to determine statistical significance. We normalized leg length by head width to correct for differences in body size. We did not use body length because body length at the first nymphal instar changes considerably with changes in food intake (well fed individuals tend to swell up). Statistical significance for the various legs and segment of the legs are summarized in Supplementary Table 4.

For the predation performance experiment, we performed a one-way ANOVA test to determine statistical significance in leg length and jump height between untreated (WT) and various RNAi experiments (Maternal-*gilt* and Maternal–Nymphal-*gilt*). We normalized leg length by body length to correct for differences in body size. We used body size because it is a reliable reference at the fifth nymphal instar. Statistical significance for differences in leg length and jump height are summarized in Supplementary Table 5.

## Additional information

**Accession codes:** The transcriptome data generated in this study have been deposited in NCBI database under the accession code PRJNA289202. *gilt* sequences generated in this study have been deposited in GenBank nucleotide database under accession codes KR704883 to KR704888.

**How to cite this article:** Armisén, D. *et al*. Predator strike shapes antipredator phenotype through new genetic interactions in water striders. *Nat. Commun.* 6:8153 doi: 10.1038/ncomms9153 (2015).

## Supplementary Material

Supplementary Figures and Tables Supplementary Figures 1-5 and Supplementary Tables 1-5

Supplementary Movie 1 Water striders are attacked by various aquatic predators. Section 1 : A water strider caught by a halfbeak fish. The fish approaches in a stealth manner from underneath the water strider and triggers a sudden and quick strike. In this video, the water strider did not react (most likely because it did not detect the attack) and was therefore caught. Section 2 : A water strider caught by a water scorpion called Ranatra (Heteroptera, Pentatomorpha). The water scorpion strikes with its raptorial forelegs wich close on the body of the water strider. Section 3 : A water strider successfully dodges the attack of a halfbeak fish. The fish strikes, but the water strider responds with a jump in the air. This results in the fish missing and the prey surviving the strike.

Supplementary Movie 2 The jump in water striders is four times faster than flight. It takes a water strider ~28 milliseconds to jump and ~120 milliseconds to fly. Therefore, the jump is a more adapted strategy to dodge the strike of aquatic predators.

Supplementary Movie 3 Water striders with shorter legs (generated by inactivating the gene *gilt*) jump lower. This video shows a snap shot of the experimental setup used to test the performance of untreated and *gilt* RNAi-treated water striders. When the jump is triggered, by the approaching predator (here, a manoeuvred dead Notonecta), gilt RNAi-treated individuals underperform compared to normal individuals. This shows how the role of *gilt* in modulating leg length contributes to increasing the chance of water striders to survive predation
