# Supplementary material for: Predator strike shapes antipredator phenotype through new genetic interactions in water striders
Source: Nat Commun. 2015 Sep 1;6:8153. doi: 10.1038/ncomms9153 (PMC4568302; doi:10.1038/ncomms9153)
Supplement: Supplementary Figures and Tables — Supplementary Figures 1-5 and Supplementary Tables 1-5 [file ncomms9153-s1.pdf]

Supplementary figure 1

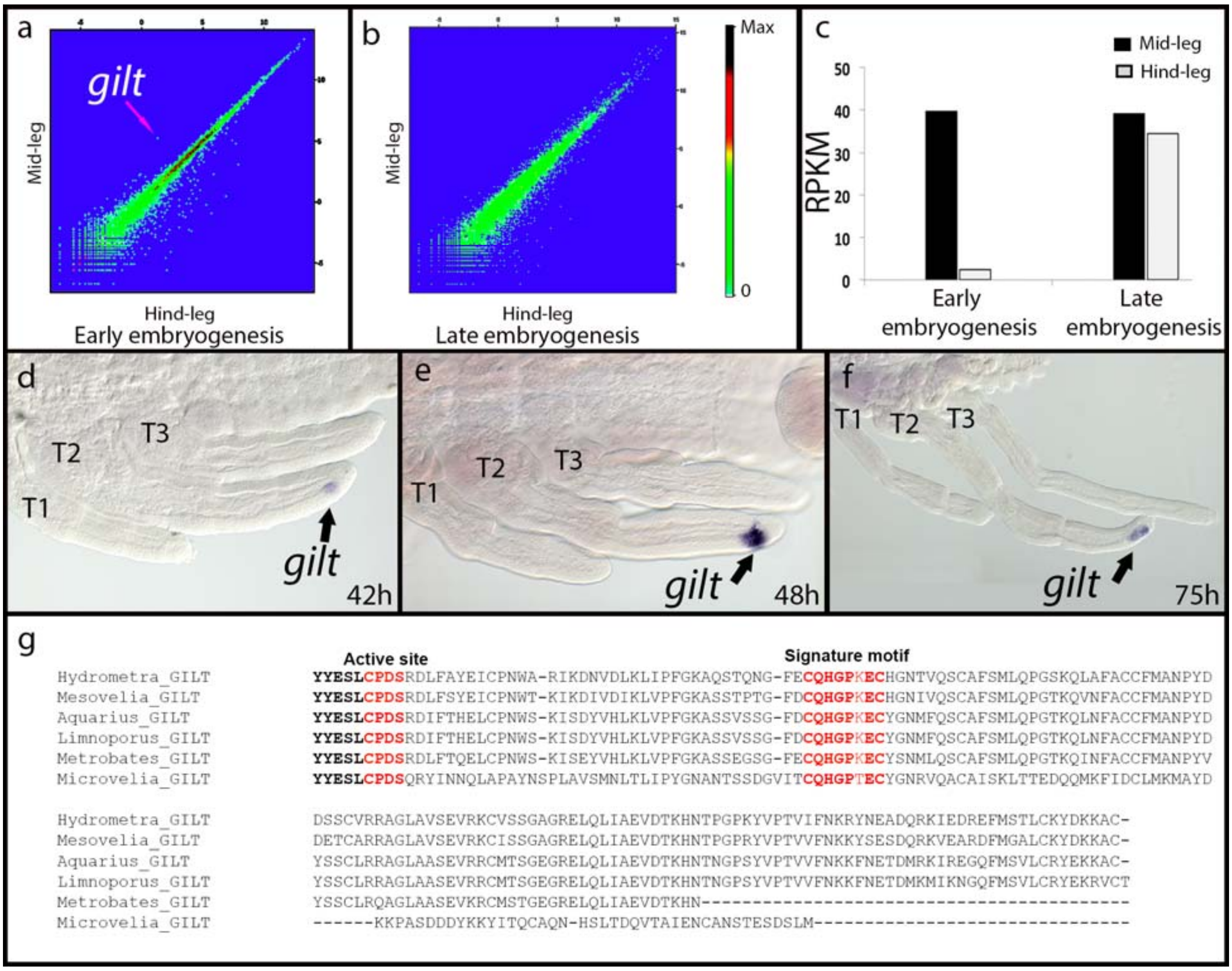

***gilt* expression during various stages of *Limnoporus* embryogenesis.** Comparative transcriptomics between mid-legs and hind-legs extracted from a pool of early (~50% to 75% embryogenesis, **a**) and late (~75% to 100% embryogenesis, **b**) embryos. *gilt* is highly expressed in mid-legs (arrow) in the early pool. (**c**) Levels of *gilt* transcript as reflected by the number of reads per kilobase per million (RPKM). Gilt expression is very high in the mid-legs early, but becomes equally expressed in both legs during late stages. (**d-f**) *gilt* mRNA appears first at 42 hours in the mid-legs, becomes strong at 48 hours, and continues in the mid-legs at 75 hours. We have not been able to detect *gilt* during late developmental stages using in situ hybridization, likely because of difficulties for probes to penetrate the cuticle that is deposited later during embryogenesis. (**g**) Alignment of Gilt protein across the six species of semi-aquatic bugs included in this study. Note the conservation of the active site and Gilt signature motif.

Supplementary figure 2:

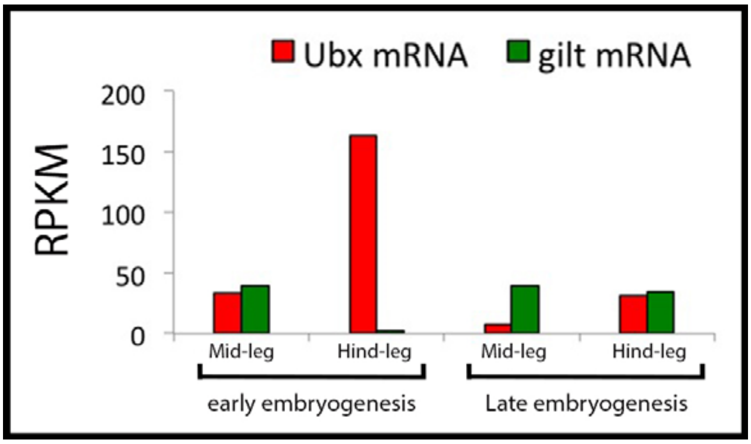

Changes in *gilt* levels (green) in relation with *Ubx* levels (red) as revealed by comparative transcriptomics between mid-legs and hind-legs during early and late embryogenesis. When Ubx level are high, *gilt* levels decrease.

Supplementary figure 3:

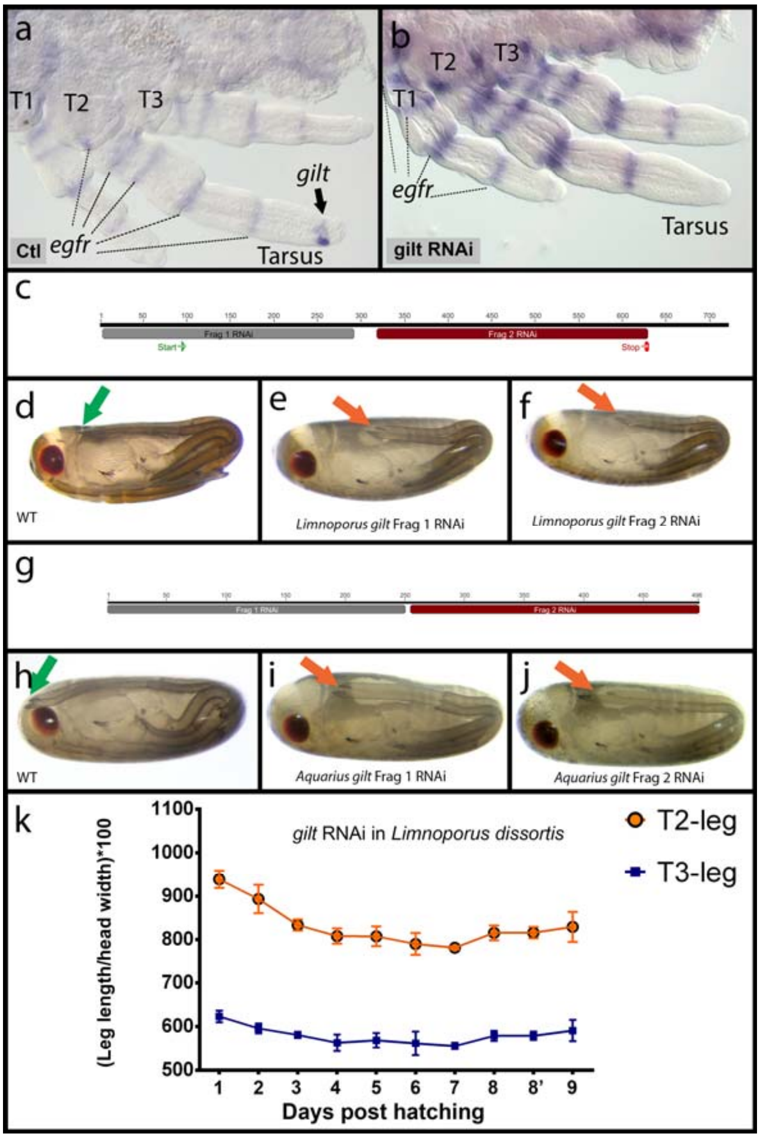

Controls for the *gilt* RNAi experiment. (**a**) Control embryo stained with a mix of *gilt* probe and ten times diluted *egfr* probe as an internal control. This probe mix detects *gilt* mRNA unambiguously in mid-leg tarsus and *egfr* mRNA in the boundaries between leg segments[12]. (**b**) *gilt* RNAi embryo stained with the same probe mix, which only detects *egfr* mRNA demonstrating that *gilt* has been successfully depleted. (**c**) A diagram presenting the two non-overlapping fragments of *Limnoporus gilt* used to synthesise double stranded RNA used in RNAi experiments. (**d**) Untreated *Limnoporus dissortis* embryo and (**e-f**) embryos obtained using the two different double stranded RNA regions of *gilt* showing the same phenotype, indicating that different fragments of *gilt* induce the same effect on leg length. (**g-h**) Two separate fragments of *gilt* in another water strider, *Aquarius paludum*, produce the same phenotype as in *Limnoporus dissortis*. (**k**) Dynamics of RNAi efficiency in *Limnoporus dissortis*. The sample size is 4 nymphs for each hatching day and error bars represent Standard Deviation. The first individuals produced after injection of double stranded RNA to the mothers tend to be either wild type or show a subtle phenotype. The strength of the phenotype increases during the following days.

Supplementary figure 4:

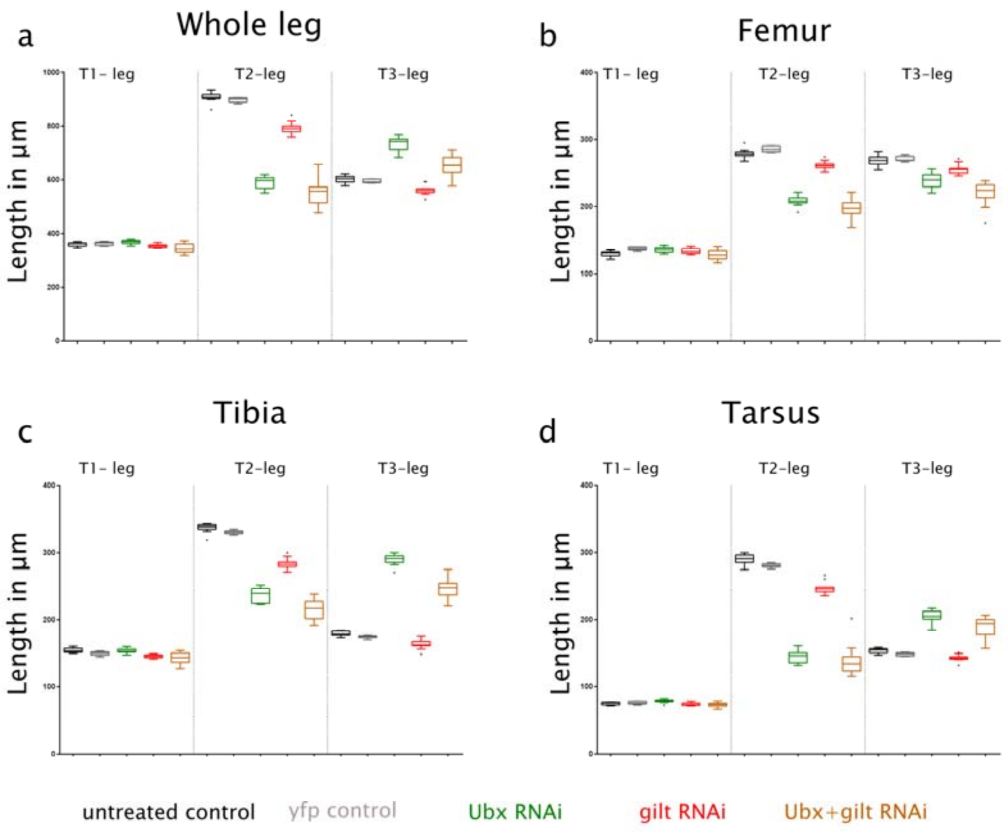

Tukey box plot showing the effect of *gilt* RNAi (n=12), *Ubx* RNAi (n=13), and *Ubx+gilt* (n=21) double RNAi on leg length and the length of the distal segments of each leg as compared to untreated controls (n=10) and yfp injected negative controls (n=7).

Supplementary figure 5: Efficiency of *gilt*, *Ubx*, and *gilt+Ubx* RNAi in *Limnoporus* embryos. (**a**) Control embryo (same as in Fig. S3a) stained with a mix of *gilt* probe and ten times diluted *egfr* probe as an internal control. This probe mix detects *gilt* mRNA unambiguously in mid-leg tarsus and *egfr* mRNA in the boundaries between leg segments[12]. (**b**) *Ubx* RNAi embryo stained with the same mix and showing unambiguous gilt mRNA expression in both mid-legs and hind-legs, along with *egfr* mRNA as an internal control. (c) *gilt* RNAi embryo (different than the one in Fig. S3b), stained with the mix of *gilt* and ten times diluted *egfr* probe, which only detects *egfr* mRNA demonstrating that *gilt* has been successfully depleted. (**d**) A double *gilt+Ubx* RNAi embryo, stained with the mix of *gilt* and ten times diluted *egfr* probe, showing a clear expression of the internal control *egfr* but no expression of *gilt* mRNA neither in the mid-legs or the hind-legs. Note in both *Ubx* RNAi and double *gilt+Ubx* RNAi the formation of an ectopic limb on the first abdominal segment (asterisk)[12].

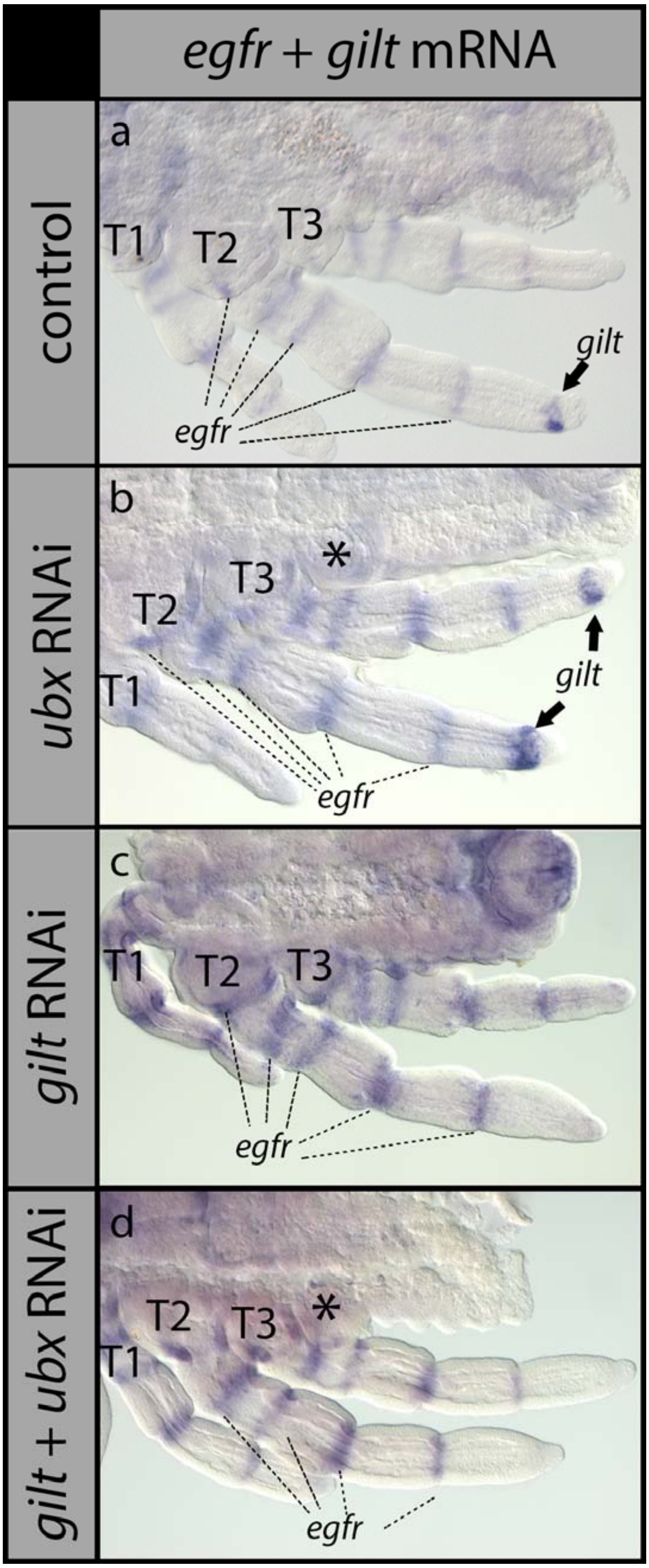

Supplementary Table 1:

	Video	Duration in micro seconds
**Halfbeak strike duration**	Halfbeak strike 1	24000
	Halfbeak strike 2	32500
	Halfbeak strike 3	27000
	Halfbeak strike 4	30000
	Halfbeak strike 5	29000
	Halfbeak strike 6	25500
	Halfbeak strike 7	35500
	Halfbeak strike 8	33000
	Halfbeak strike 9	25000
	Halfbeak strike 10	26000
	**Average**	**28750**
***Limnoporus* jump duration**	Jump 1	30500
	Jump 2	29000
	Jump 3	27000
	Jump 4	27000
	Jump 5	32000
	Jump 6	29500
	Jump 7	28500
	Jump 8	29000
	Jump 9	26000
	Jump 10	30000
	**Average**	**28850**
***Limnoporus* flight duration**	Flight 1	112000
	Flight 2	152500
	Flight 3	96000
	Flight 4	131000
	Flight 5	124500
	Flight 6	126000
	Flight 7	145000
	Flight 8	110000
	Flight 9	107000
	Flight 10	144000
	**Average**	**124800**

Duration of strike in halfbeak fish, jump and flight in adult water striders calculated as an average of ten strikes, jumps and flight events respectively.

Supplementary table 2:

Species	Forward	Reverse
Limnoporus dissortis	AAGCCCCTACTATCAATGCCCAATG	TCAGCAGACTCTCTTCTCGTATCGG
Aquarius paludum	TACTACGAGTCGCTCTGTCCAGACAGC	CAGCAGACTCTCTTGTCGTATCGGCAGA
Hydrometra stagnorum	TACTACGAGTCACTTGCCCTGACA	TAGCAGGCCTTTTTGTCGTACTTGC
Metrobates hesperius	TACTACGAGTCGCTCTGTCCAGACAGC	GTGTTGTGTTTGGTGTCTACTTCAG
Microvelia americana	TTTGCGGGTTTCTACCAGCACTTT	GCCATGAGAGAATCAGATTCAGTGC
Mesovelia mulsanti	TACTACGAGTCACTCTGCCCTGACA	TAGCAGGCCTTTTTGTCGTACTTGC

Primer sequences used to clone *gilt* from the six semi-aquatic bugs species presented in this study.

Supplementary table 3:

		Forward	Reverse
Limnoporus dissortis	complete	taatacgactcactatagggagacca cAAGCCCCTACTATCAATGCCCAATG	M13-R_T7
	Frag 1	taatacgactcactatagggagacca cAAGCCCCTACTATCAATGCCCAATG	taatacgactcactatagggagacca cCATTCTTTCGGACCATGCTGACA
	Frag 2	taatacgactcactatagggagacca cCTTTCAGCATGCTGCAACCTGG	taatacgactcactatagggagacca cTCAGCAGACTCTCTTCTCGTATCGG
Aquarius paludum	Frag 1	taatacgactcactatagggagacca cTACTACGAGTCGCTATGCCCTGACA GC	taatacgactcactatagggagacca cGGTTTGCCATGAAACAGCAGGC
	Frag 2	taatacgactcactatagggagacca cCGACTACAGTTCATGTCTAAGAAG	taatacgactcactatagggagacca cCACAGGCTTTTTTCTCGTACCTGCA

Primers used to synthesize double stranded RNA used in RNAi experiments in *Limnoporus* and *Aquarius*. These primers are tagged with T7 promoter (small case letters) to enable in vitro transcription using the T7 polymerase enzyme.

Supplementary table 4:

		1way ANOVA (T1 leg Femur)	Mean 1	Mean 2	Mean Diff,	95% CI of diff,	Significant?	Summary	Adjusted P Value
Fore-legs	Femur	WT vs. Control	129,9	137,1	-7,191	-14,52 to 0,1379	No	ns	0,0569
		WT vs. Ubx+GILT	129,9	128,3	1,604	-4,110 to 7,319	No	ns	0,9323
		WT vs. GILT	129,9	133,6	-3,666	-10,03 to 2,702	No	ns	0,4905
		WT vs. Ubx	129,9	135,6	-5,636	-11,89 to 0,6196	No	ns	0,0965
		Control vs. Ubx+GILT	137,1	128,3	8,796	2,305 to 15,29	Yes	**	0,003
		Control vs. GILT	137,1	133,6	3,525	-3,548 to 10,60	No	ns	0,6281
		Control vs. Ubx	137,1	135,6	1,555	-5,417 to 8,527	No	ns	0,9699
		Ubx+GILT vs. GILT	128,3	133,6	-5,27	-10,65 to 0,1114	No	ns	0,0577
		Ubx+GILT vs. Ubx	128,3	135,6	-7,24	-12,49 to -1,992	Yes	**	0,0024
		GILT vs. Ubx	133,6	135,6	-1,97	-7,924 to 3,984	No	ns	0,8834
	Tibia	WT vs. Control	153,7	149,4	4,353	-3,028 to 11,73	No	ns	0,4661
		WT vs. Ubx+GILT	153,7	142,5	11,2	5,446 to 16,95	Yes	****	< 0,0001
		WT vs. GILT	153,7	145,4	8,355	1,942 to 14,77	Yes	**	0,0047
		WT vs. Ubx	153,7	153,7	0,0245	-6,275 to 6,324	No	ns	> 0,9999
		Control vs. Ubx+GILT	149,4	142,5	6,848	0,3109 to 13,38	Yes	*	0,0356
		Control vs. GILT	149,4	145,4	4,002	-3,121 to 11,13	No	ns	0,5148
		Control vs. Ubx	149,4	153,7	-4,328	-11,35 to 2,693	No	ns	0,4208
		Ubx+GILT vs. GILT	142,5	145,4	-2,845	-8,265 to 2,575	No	ns	0,5807
		Ubx+GILT vs. Ubx	142,5	153,7	-11,18	-16,46 to -5,890	Yes	****	< 0,0001
		GILT vs. Ubx	145,4	153,7	-8,33	-14,33 to -2,335	Yes	**	0,0022
	Tarsus	WT vs. Control	75,36	76,11	-0,7419	-4,227 to 2,744	No	ns	0,9747
		WT vs. Ubx+GILT	75,36	73,31	2,056	-0,6619 to 4,773	No	ns	0,2217
		WT vs. GILT	75,36	74,03	1,332	-1,697 to 4,360	No	ns	0,7293
		WT vs. Ubx	75,36	78,77	-3,404	-6,379 to -0,4289	Yes	*	0,0172
		Control vs. Ubx+GILT	76,11	73,31	2,797	-0,2895 to 5,884	No	ns	0,0934
		Control vs. GILT	76,11	74,03	2,074	-1,290 to 5,437	No	ns	0,4208
		Control vs. Ubx	76,11	78,77	-2,662	-5,978 to 0,6538	No	ns	0,1728
		Ubx+GILT vs. GILT	73,31	74,03	-0,7237	-3,283 to 1,836	No	ns	0,9307
		Ubx+GILT vs. Ubx	73,31	78,77	-5,459	-7,955 to -2,963	Yes	****	< 0,0001
		GILT vs. Ubx	74,03	78,77	-4,736	-7,567 to -1,904	Yes	***	0,0002
	Total	WT vs. Control	359,5	362,6	-3,073	-19,05 to 12,90	No	ns	0,9825
		WT vs. Ubx+GILT	359,5	344,1	15,37	2,911 to 27,82	Yes	**	0,0084
		WT vs. GILT	359,5	353	6,528	-7,354 to 20,41	No	ns	0,6776
		WT vs. Ubx	359,5	368	-8,508	-22,15 to 5,129	No	ns	0,4084
		Control vs. Ubx+GILT	362,6	344,1	18,44	4,291 to 32,59	Yes	**	0,0046
		Control vs. GILT	362,6	353	9,601	-5,818 to 25,02	No	ns	0,4105
		Control vs. Ubx	362,6	368	-5,435	-20,63 to 9,764	No	ns	0,8512
		Ubx+GILT vs. GILT	344,1	353	-8,839	-20,57 to 2,893	No	ns	0,2252
		Ubx+GILT vs. Ubx	344,1	368	-23,88	-35,32 to -12,43	Yes	****	< 0,0001
		GILT vs. Ubx	353	368	-15,04	-28,01 to -2,057	Yes	*	0,0154
Mid-legs	Femur	WT vs. Control	279,1	285,9	-6,752	-19,53 to 6,026	No	ns	0,5745
		WT vs. Ubx+GILT	279,1	197,9	81,15	71,19 to 91,12	Yes	****	< 0,0001
		WT vs. GILT	279,1	262	17,08	5,977 to 28,18	Yes	***	0,0006
		WT vs. Ubx	279,1	208,2	70,93	60,02 to 81,84	Yes	****	< 0,0001
		Control vs. Ubx+GILT	285,9	197,9	87,91	76,59 to 99,22	Yes	****	< 0,0001
		Control vs. GILT	285,9	262	23,83	11,50 to 36,16	Yes	****	< 0,0001
		Control vs. Ubx	285,9	208,2	77,68	65,53 to 89,84	Yes	****	< 0,0001
		Ubx+GILT vs. GILT	197,9	262	-64,07	-73,46 to -54,69	Yes	****	< 0,0001
		Ubx+GILT vs. Ubx	197,9	208,2	-10,22	-19,37 to -1,072	Yes	*	0,0212
		GILT vs. Ubx	262	208,2	53,85	43,47 to 64,23	Yes	****	< 0,0001
	Tibia	WT vs. Control	335,8	330,8	5,018	-10,02 to 20,05	No	ns	0,8802
		WT vs. Ubx+GILT	335,8	214,9	120,9	109,2 to 132,7	Yes	****	< 0,0001
		WT vs. GILT	335,8	283,5	52,34	39,28 to 65,41	Yes	****	< 0,0001
		WT vs. Ubx	335,8	236,8	99,06	86,22 to 111,9	Yes	****	< 0,0001
		Control vs. Ubx+GILT	330,8	214,9	115,9	102,6 to 129,2	Yes	****	< 0,0001
		Control vs. GILT	330,8	283,5	47,33	32,82 to 61,84	Yes	****	< 0,0001
		Control vs. Ubx	330,8	236,8	94,04	79,73 to 108,3	Yes	****	< 0,0001
		Ubx+GILT vs. GILT	214,9	283,5	-68,59	-79,63 to -57,55	Yes	****	< 0,0001
		Ubx+GILT vs. Ubx	214,9	236,8	-21,88	-32,65 to -11,12	Yes	****	< 0,0001
		GILT vs. Ubx	283,5	236,8	46,71	34,50 to 58,92	Yes	****	< 0,0001
	Tarsus	WT vs. Control	289,1	280,5	8,649	-9,181 to 26,48	No	ns	0,6517
		WT vs. Ubx+GILT	289,1	137,3	151,8	137,9 to 165,7	Yes	****	< 0,0001
		WT vs. GILT	289,1	247,3	41,88	26,39 to 57,37	Yes	****	< 0,0001
		WT vs. Ubx	289,1	144,4	144,7	129,5 to 160,0	Yes	****	< 0,0001
		Control vs. Ubx+GILT	280,5	137,3	143,2	127,4 to 159,0	Yes	****	< 0,0001
		Control vs. GILT	280,5	247,3	33,23	16,02 to 50,44	Yes	****	< 0,0001
		Control vs. Ubx	280,5	144,4	136,1	119,1 to 153,0	Yes	****	< 0,0001
		Ubx+GILT vs. GILT	137,3	247,3	-110	-123,0 to -96,86	Yes	****	< 0,0001
		Ubx+GILT vs. Ubx	137,3	144,4	-7,102	-19,87 to 5,666	No	ns	0,5248
		GILT vs. Ubx	247,3	144,4	102,9	88,37 to 117,3	Yes	****	< 0,0001
	Total	WT vs. Control	904,1	897,2	6,915	-34,28 to 48,11	No	ns	0,9895
		WT vs. Ubx+GILT	904,1	550,1	353,9	321,8 to 386,0	Yes	****	< 0,0001
		WT vs. GILT	904,1	792,8	111,3	75,51 to 147,1	Yes	****	< 0,0001
		WT vs. Ubx	904,1	589,4	314,7	279,6 to 349,9	Yes	****	< 0,0001
		Control vs. Ubx+GILT	897,2	550,1	347	310,5 to 383,5	Yes	****	< 0,0001
		Control vs. GILT	897,2	792,8	104,4	64,63 to 144,1	Yes	****	< 0,0001
		Control vs. Ubx	897,2	589,4	307,8	268,6 to 347,0	Yes	****	< 0,0001

		Ubx+GILT vs. GILT	550,1	792,8	-242,6	-272,9 to -212,4	Yes	****	< 0,0001
		Ubx+GILT vs. Ubx	550,1	589,4	-39,21	-68,71 to -9,707	Yes	**	0,0037
		GILT vs. Ubx	792,8	589,4	203,4	169,9 to 236,9	Yes	****	< 0,0001
Hind-legs	Femur	WT vs. Control	268,5	271,5	-3,009	-18,55 to 12,53	No	ns	0,9821
		WT vs. Ubx+GILT	268,5	220	48,55	36,43 to 60,66	Yes	****	< 0,0001
		WT vs. GILT	268,5	255,9	12,64	-0,8627 to 26,14	No	ns	0,077
		WT vs. Ubx	268,5	239,2	29,29	16,03 to 42,55	Yes	****	< 0,0001
		Control vs. Ubx+GILT	271,5	220	51,56	37,80 to 65,32	Yes	****	< 0,0001
		Control vs. GILT	271,5	255,9	15,65	0,6513 to 30,64	Yes	*	0,0367
		Control vs. Ubx	271,5	239,2	32,3	17,52 to 47,08	Yes	****	< 0,0001
		Ubx+GILT vs. GILT	220	255,9	-35,91	-47,32 to -24,50	Yes	****	< 0,0001
		Ubx+GILT vs. Ubx	220	239,2	-19,26	-30,39 to -8,130	Yes	****	< 0,0001
		GILT vs. Ubx	255,9	239,2	16,65	4,030 to 29,28	Yes	**	0,0041
	Tibia	WT vs. Control	178,7	174	4,692	-9,355 to 18,74	No	ns	0,8799
		WT vs. Ubx+GILT	178,7	248,6	-69,85	-80,80 to -58,90	Yes	****	< 0,0001
		WT vs. GILT	178,7	163	15,66	3,456 to 27,87	Yes	**	0,0055
		WT vs. Ubx	178,7	289,8	-111,1	-123,1 to -99,08	Yes	****	< 0,0001
		Control vs. Ubx+GILT	174	248,6	-74,54	-86,98 to -62,10	Yes	****	< 0,0001
		Control vs. GILT	174	163	10,97	-2,587 to 24,53	No	ns	0,1668
		Control vs. Ubx	174	289,8	-115,8	-129,1 to -102,4	Yes	****	< 0,0001
		Ubx+GILT vs. GILT	248,6	163	85,51	75,20 to 95,83	Yes	****	< 0,0001
		Ubx+GILT vs. Ubx	248,6	289,8	-41,22	-51,28 to -31,16	Yes	****	< 0,0001
		GILT vs. Ubx	163	289,8	-126,7	-138,1 to -115,3	Yes	****	< 0,0001
	Tarsus	WT vs. Control	153,1	148,4	4,713	-8,410 to 17,84	No	ns	0,8493
		WT vs. Ubx+GILT	153,1	188,5	-35,39	-45,62 to -25,15	Yes	****	< 0,0001
		WT vs. GILT	153,1	142,6	10,5	-0,8973 to 21,91	No	ns	0,0848
		WT vs. Ubx	153,1	204,9	-51,83	-63,03 to -40,63	Yes	****	< 0,0001
		Control vs. Ubx+GILT	148,4	188,5	-40,1	-51,72 to -28,48	Yes	****	< 0,0001
		Control vs. GILT	148,4	142,6	5,792	-6,873 to 18,46	No	ns	0,6998
		Control vs. Ubx	148,4	204,9	-56,55	-69,03 to -44,06	Yes	****	< 0,0001
		Ubx+GILT vs. GILT	188,5	142,6	45,89	36,25 to 55,53	Yes	****	< 0,0001
		Ubx+GILT vs. Ubx	188,5	204,9	-16,45	-25,84 to -7,050	Yes	****	< 0,0001
		GILT vs. Ubx	142,6	204,9	-62,34	-73,00 to -51,68	Yes	****	< 0,0001
	Total	WT vs. Control	600,3	594	6,395	-31,17 to 43,96	No	ns	0,989
		WT vs. Ubx+GILT	600,3	657	-56,69	-85,97 to -27,40	Yes	****	< 0,0001
		WT vs. GILT	600,3	561,5	38,8	6,168 to 71,44	Yes	*	0,012
		WT vs. Ubx	600,3	734	-133,6	-165,7 to -101,6	Yes	****	< 0,0001
		Control vs. Ubx+GILT	594	657	-63,08	-96,35 to -29,82	Yes	****	< 0,0001
		Control vs. GILT	594	561,5	32,41	-3,842 to 68,66	No	ns	0,1008
		Control vs. Ubx	594	734	-140	-175,7 to -104,3	Yes	****	< 0,0001
		Ubx+GILT vs. GILT	657	561,5	95,49	67,91 to 123,1	Yes	****	< 0,0001
		Ubx+GILT vs. Ubx	657	734	-76,92	-103,8 to -50,02	Yes	****	< 0,0001
		GILT vs. Ubx	561,5	734	-172,4	-202,9 to -141,9	Yes	****	< 0,0001

Supplementary table 4: Statistical analyses and significance of the effect of various RNAi experiments on leg length.

Statistical analyses and significance of the effect of various RNAi experiments on leg length.

Supplementary table 5:

1way ANOVA (Jumps)	Mean 1	Mean 2	Mean Diff,	95% CI of diff,	Significant?	Summary	Adjusted P Value
WT vs. M-*gilt*	5,050	4,200	0,8500	0,4261 to 1,274	Yes	****	< 0,0001
WT vs. M-N_*gilt*	5,050	4,050	1,000	0,5544 to 1,446	Yes	****	< 0,0001
M-*gilt* vs. M-N-*gilt*	4,200	4,050	0,1500	-0,2739 to 0,5739	No	ns	0,7859

One-way ANOVA analyses and significance of the effect of *gilt* RNAi on the jump performance.
